# Examination of Intracellular GPCR-Mediated Signaling with High Temporal Resolution

**DOI:** 10.3390/ijms23158516

**Published:** 2022-07-31

**Authors:** Nadine Gruteser, Arnd Baumann

**Affiliations:** Institute of Biological Information Processing, IBI-1, Research Center Jülich, 52428 Jülich, Germany; nadine.gruteser@t-online.de

**Keywords:** biogenic amines, caged compounds, cell-based functional assays, cyclic adenosine monophosphate, Ca^2+^ imaging, stopped-flow measurements

## Abstract

The GTP-binding protein-coupled receptors (GPCRs) play important roles in physiology and neuronal signaling. More than a thousand genes, excluding the olfactory receptors, have been identified that encode these integral membrane proteins. Their pharmacological and functional properties make them fascinating targets for drug development, since various disease states can be treated and overcome by pharmacologically addressing these receptors and/or their downstream interacting partners. The activation of the GPCRs typically causes transient changes in the intracellular second messenger concentrations as well as in membrane conductance. In contrast to ion channel-mediated electrical signaling which results in spontaneous cellular responses, the GPCR-mediated metabotropic signals operate at a different time scale. Here we have studied the kinetics of two common GPCR-induced signaling pathways: (a) Ca^2+^ release from intracellular stores and (b) cyclic adenosine monophosphate (cAMP) production. The latter was monitored via the activation of cyclic nucleotide-gated (CNG) ion channels causing Ca^2+^ influx into the cell. Genetically modified and stably transfected cell lines were established and used in stopped-flow experiments to uncover the individual steps of the reaction cascades. Using two homologous biogenic amine receptors, either coupling to G_o/q_ or G_s_ proteins, allowed us to determine the time between receptor activation and signal output. With ~350 ms, the release of Ca^2+^ from intracellular stores was much faster than cAMP-mediated Ca^2+^ entry through CNG channels (~6 s). The measurements with caged compounds suggest that this difference is due to turnover numbers of the GPCR downstream effectors rather than the different reaction cascades, per se.

## 1. Introduction

Uni- and multicellular organisms continuously register and react to external and internal signals. These signals typically evoke specific cellular responses by activating signal transduction processes that often cause transient changes in the intracellular second-messenger concentrations. A prominent class of signal-detecting proteins are metabotropic G protein-coupled receptors (GPCRs), whose activation result in, e.g., the intracellular release of Ca^2+^ ions from internal stores or changes in the concentration of cyclic adenosine monophosphate (cAMP) (for reviews see [1,2,3]). G protein-coupled receptors comprise the largest gene family of membrane receptors in the human body and serve as drug targets for at least one third of the pharmaceuticals marketed worldwide [4,5]. Based on their sequence homology, the GPCRs have been categorized into five main families with rhodopsin-like receptors forming the largest group and representing ca. 80% of the human GPCRs [6].

A cognate feature of GPCRs is their shared transmembrane topography. The proteins consist of seven membrane-spanning segments connected by three extracellular and three intracellular loops. The N-terminus is placed on the extracellular side, whereas the C-terminus of the protein is located intracellularly [7,8]. The binding of a ligand to the receptor causes a structural change in the molecule that is conveyed to the intracellular loops. Here, the activated receptor stimulates the associated trimeric GTP-binding (G) proteins [9,10]. Interaction between the receptor and G-protein leads to an exchange reaction in the α subunit of the G-protein. Bound guanosine-diphosphate (GDP) is released and substituted by guanosine-triphosphate (GTP). The GTP-bound α subunit (Gα_GTP_) dissociates from the β/γ complex of the G-protein and interacts with downstream effectors that finally cause changes, e.g., in the cyclic nucleotide concentrations [cNMP]_i_ or [Ca^2+^]_i_. Once a ligand has bound to the GPCR, each activated receptor may stimulate up to a few hundreds of G-proteins before the receptor becomes desensitized [11,12,13]. The Gα subunit harbors an intrinsic GTPase function and hydrolyzes the bound GTP to GDP. In the GDP-bound form, the Gα subunit re-associates with the Gβ/γ complex forming an inactive G-protein which can be activated by a newly stimulated GPCR.

One important group of ligands that bind to the GPCRs of the rhodopsin-like family are biogenic amines [14]. In both vertebrates and invertebrates, five biogenic amines are known. Besides molecules acting in both phylogenetic groups (dopamine, histamine, and serotonin), other biogenic amines preferentially act in vertebrates (norepinephrine, epinephrine) or invertebrates (tyramine, octopamine) (for reviews see [15,16]). High concentrations of octopamine and tyramine have been found in insects’ nervous tissues, but only traces in vertebrate brains [17,18]. Octopamine is a structural analogue of norepinephrine and functionally considered as a “fight or flight” hormone. The physiological functions of the octopamine receptors in invertebrates can be compared to those of the adrenergic receptors in the vertebrate sympathetic nervous system [19]. Several genes have been characterized in invertebrate model organisms, e.g., *Drosophila melanogaster, Apis mellifera*, or *Periplaneta americana*, encoding the functional octopamine receptors. In *D. melanogaster*, an alternatively spliced gene codes for the receptors activating phospholipase C (PLC), which leads to the IP_3_-mediated Ca^2+^ release from internal stores (DmOctα1A/1B R; [20,21]. Three receptor genes were identified that cause the activation of adenylyl cyclase, and thus the production of cAMP (DmOctβ1-3R; [21,22]). More recently, a fifth gene was uncovered encoding a receptor that inhibits cAMP production (DmOctα2R; [23]). The family of the octopamine receptors expressed in *A. mellifera* is similarily complex. The AmOctα1R causes the Ca^2+^ release from intracellular stores [24]. The AmOctα2R inhibits the production of cAMP [25], and, finally, the receptors AmOctß1-4R stimulate the cAMP production [26]. In the cockroach, only two receptors have been functionally examined, so far. The PaOctα1R causes the Ca^2+^ release from intracellular stores [27], whereas the PaOctβ2R leads to the cAMP production [28]. In addition to examining intracellular-coupling pathways for each of these receptors, the pharmacological properties of the proteins were quite extensively assessed. However, the experimental data on the kinetics of the cellular signal transduction events have not so far been collected.

We decided to address this issue using genetically modified cell lines, and to analyze the time course from the receptor activation to the signal development with high temporal resolution, using a stopped-flow apparatus. Since we were interested in the GPCR pathways either evoking cAMP or Ca^2+^ signals, we used the DmOctβ1R and the DmOctα1B receptor-encoding genes to generate the stably transfected cell lines. For the signal read out, Ca^2+^ imaging with Fluo-4 [29] and the genetically encoded Ca^2+^ indicator, GCaMP3.0 [30], were used. Since DmOctβ1R does not cause the Ca^2+^ signals per se, the cell line was equipped with a gene encoding a cyclic nucleotide-gated (CNG) ion channel [31,32], that opens upon binding to cAMP and mediates the Ca^2+^ influx from the extracellular space. We provide evidence that this strategy: (a) allowed for the measurement of the time-resolved signal generation with intact cells; (b) allowed the analyses of individual transduction steps employing, e.g., caged compounds; and (c) supports the notion that the cAMP- and Ca^2+^-mediated transduction pathways operate at distinct time scales, most likely due to the biochemical properties of the participating effector enzymes.

## 2. Results

### 2.1. Establishing Stably Transfected Cell Lines for Kinetic Measurements

In order to investigate the kinetics of GPCR-mediated signaling, HEK293 cell lines were used, constitutively expressing either DmOctα1B or DmOctβ1 octopamine receptors from *D. melanogaster* [21]. The activation of DmOctα1B receptors leads to an increase in [Ca^2+^]_i_, via a PLC-mediated pathway, whereas the stimulation of DmOctβ1 leads to activation of the membrane-bound ACs and thereby triggers an increase in [cAMP]_i_ (Figure S1, Supplementary Materials) [21].

Upon activation of the DmOctα1B receptors, the intracellular Ca^2+^ signals were monitored with Fluo-4 [29] and the genetically encoded calcium indicator (GECI) GCaMP3.0 [30]. Both of the detectors respond to the binding of Ca^2+^ with an increase in the fluorescence intensity. To monitor the changes in [cAMP]_i_ mediated by the DmOctβ1 receptors, a HEK293 cell line constitutively expressing the receptor as well as a cAMP-sensitive variant of the A2 subunit of the bovine olfactory cyclic nucleotide-gated ion channel (CNGA2; flpTM cells) [32] was used.

Both the DmOctα1B receptor- and DmOctβ1 receptor-expressing (=flpTM-DmOctβ1) cell lines were initially investigated via Ca^2+^ fluorimetric measurements in a plate reader. The cells were seeded in 96 well plates and loaded with Fluo-4, enabling the detection of changes in [Ca^2+^]_i_. The Fluo-4 was excited at 485 nm and the fluorescence emission was recorded at 510 nm. To overcome the cAMP degradation by cell-endogenous phosphodiesterases, the DmOctβ1-expressing cells were treated with 100 µM iso-butylmethylxanthine (IBMX). Prior to the ligand application, the basal fluorescence (F_0_) was recorded and the concentration–response curves were generated by plotting the changes in fluorescence ((F_max_ − F_0_)/F_0_ = ΔF/F_0_) against the ligand concentrations. For a better comparison, the fluorescence intensities were normalized. The maximal change in fluorescence for each of the stimulation series was defined as 100%. The EC_50_ values were determined from a nonlinear regression plot (four parameters) using GraphPad Prism v5.04 for analysis and display. The calculated EC_50_ for octopamine was ~12 nM on DmOctα1B and ~1.2 nM on DmOctβ1.

**Figure 1:**
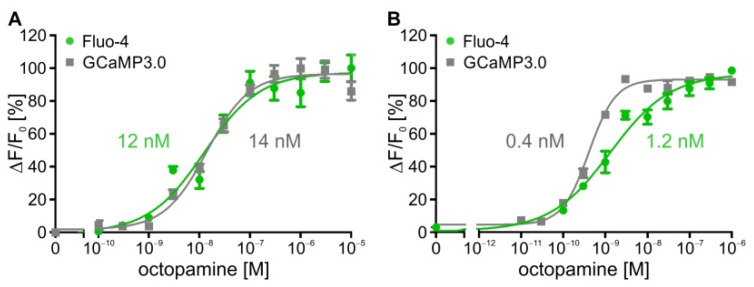
Concentration–response curves for octopamine on DmOctα1B and flpTM-DmOctβ1 cells loaded with Fluo-4 or expressing GCaMP3.0. (**A**) Human embryonic kidney cells (HEK293) constitutively expressing DmOctα1B receptors were either loaded with Fluo-4 (green) or stably transfected with GCaMP3.0 (grey); (**B**) Human embryonic kidney cells (HEK293) constitutively expressing DmOctβ1 receptors and CNGA2 channels (flpTM-DmOctβ1) were either loaded with Fluo-4 (green) or stably transfected with GCaMP3.0 (grey). Concentration-dependent effects of octopamine on [Ca^2+^]_i_ were determined. Relative change in fluorescence (ΔF/F_0_) is given as the percentage of the value obtained with the highest octopamine concentration (=100%). Octopamine activation of both receptors led to a concentration-dependent increase in the fluorescence signal. The increase in [Ca^2+^]_i_ in flpTM-DmOctβ1 cells is due to receptor-mediated production of cAMP and subsequent opening of CNGA2 channels causing Ca^2+^ influx into the cell. During measurements with flpTM-DmOctβ1 cells, cAMP degradation was inhibited with 100 µM IBMX. A total of five independent measurements were performed. Data points represent the mean ± SD of a representative eight-fold determination.

In order to omit loading with the fluorescent dye, both the DmOctα1B-expressing cells and flpTM-DmOctβ1 cells were stably transfected with a construct encoding the cytosolic version of GCaMP3.0. As in the previous experiment with Fluo-4, the GCaMP3.0-expressing cell lines were seeded in 96 well plates and incubated with the same series of increasing octopamine concentrations. The GCaMP3.0 was excited at 485 nm and the fluorescence emission was recorded at 510 nm. The basal fluorescence (F_0_) was recorded before adding the octopamine concentration series. The fluorescence intensities were normalized to the maximal change in fluorescence, defined as 100%. The concentration–response curves were generated with GraphPad Prism v5.04. In Figure 1, the concentration–response curves obtained with Fluo-4 and GCaMP3.0 are depicted for both of the receptors. With EC_50_ values of 12 nM (Fluo-4) and 14 nM (GCaMP3.0) the results for the DmOctα1B cell lines were very similar (Figure 1A). The corresponding EC_50_ values for DmOctβ1-expressing cell lines (Figure 1B) were an order of magnitude smaller with 1.2 nM (Fluo-4) and 0.4 nM (GCaMP3.0). Thus, the experimental regime enabled monitoring of intracellular changes of [Ca^2+^] either directly, via DmOctα1B, or indirectly, via DmOctβ1-dependent cAMP-mediated opening of CNGA2, equally well, independent of employing Fluo-4 or GCaMP3.0 as detectors. Nevertheless, the EC_50_ values of both receptors for octopamine differed approximately by an order of magnitude.

**Figure 2:**
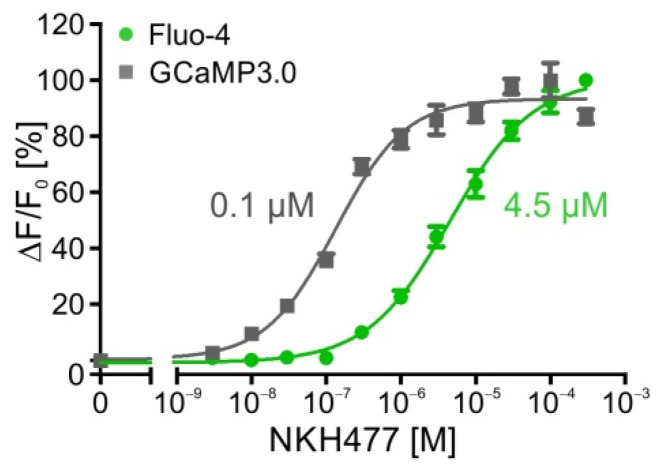
Stimulation of DmOctβ1-expressing cells with NKH477. Human embryonic kidney cells (HEK293) constitutively expressing DmOctβ1 receptors and CNGA2 channels (flpTM-DmOctβ1) were either loaded with Fluo-4 (green) or stably transfected with GCaMP3.0 (grey). Concentration-dependent effects of NKH477-mediated activation of cell endogenous adenylyl cyclases on [Ca^2+^]_i_ were determined. Relative change in fluorescence (ΔF/F_0_) is given as the percentage of the value obtained with the highest NKH477 concentration (=100%). Application of NKH477 led to concentration-dependent increases in the fluorescence signal originating from adenylyl cyclase activation and subsequent opening of CNGA2 channels causing Ca^2+^ influx into the cell. During measurements, cAMP degradation was inhibited with 100 µM IBMX. A total of five independent measurements were performed. Data points represent the mean ± SD of a representative eight-fold determination.

In another series of experiments, DmOctβ1 receptor-expressing cell lines were incubated with NKH477, a water soluble agonist of membrane bound adenylyl cyclases, the downstream effectors in DmOctβ1 signaling. Before stimulating the adenylyl cyclase activity, NKH477 has to cross the cell membrane and then binds to the catalytic domain of the enzyme. As before, the flpTM-DmOctβ1 cells were seeded in 96 MWPs and loaded with Fluo-4. In parallel, cells stably co-transfected with GCaMP3.0 were examined (flpTM-DmOctβ1-GCaMP3.0). All of the measurements were performed in the presence of 100 µM IBMX. Figure 2 shows the concentration–response curves of the flpTM-DmOctβ1 cells loaded with Fluo-4 and flpTM-DmOctβ1-GCaMP3.0 cells for NKH477. In comparison to the octopamine-evoked responses, the EC_50_ values for NKH477 differed strongly, with values in the nanomolar range for octopamine and in the micromolar range for NKH477, but confirming the previous data on the octopamine receptors and adenylyl cyclases, respectively [21,22,32]. Notably, the EC_50_ values determined with GCaMP3.0 were about one order of magnitude lower than those obtained with Fluo-4. Whether this effect was due to differences in the cytosolic distribution of both Ca^2+^ detectors and/or the spatial presence of adenylyl cyclase isoforms in membrane domains has not been experimentally addressed. The EC_50_ values for octopamine and NKH477 are listed in Table 1.

Having proven that the cell lines were suited and reacted to stimuli in a predictable way, we set out to use them in the stopped-flow experiments.

### 2.2. Time-Resolved Measurements with DmOctα1B-Expressing Cells

The assays performed so far were conducted with cells adhering to the bottom of a multi well plate, supporting the typical adhesive behavior of HEK293 cells. For measurements in a stopped-flow system, the cells in suspension and ligand solutions are quickly mixed in the apparatus and then conveyed to the optical unit where the fluorescence signals were measured. In order to gain information about the survival rate of HEK293 cells in the stopped-flow system, the cell suspensions were applied with different flow rates (0.5, 1.0 and 2.0 mL) and the cells were collected at the outlet of the apparatus. They were transferred into a petri dish and cultivated for 24 h. All of the flow rates resulted in survival of ≥80% of introduced cells. A flow rate of 1 mL was finally chosen to guarantee high survival rates (~95%) in combination with short dead times (36 ms) of the system.

The control experiments were also performed to examine whether ligand-independent Ca^2+^ signals occurred over time. The HEK293 cells, either loaded with Fluo-4 or constitutively expressing GCaMP3.0, were monitored in the stopped-flow apparatus by recording the fluorescence signals every 5 min for a total of 60 min. A slight increase in the basal fluorescence was registered over time for the loaded Fluo-4 cells as well as for the GCaMP3.0-expressing cells, but this intensity did not compromise the ligand induced signals (see below).

**Figure 3:**
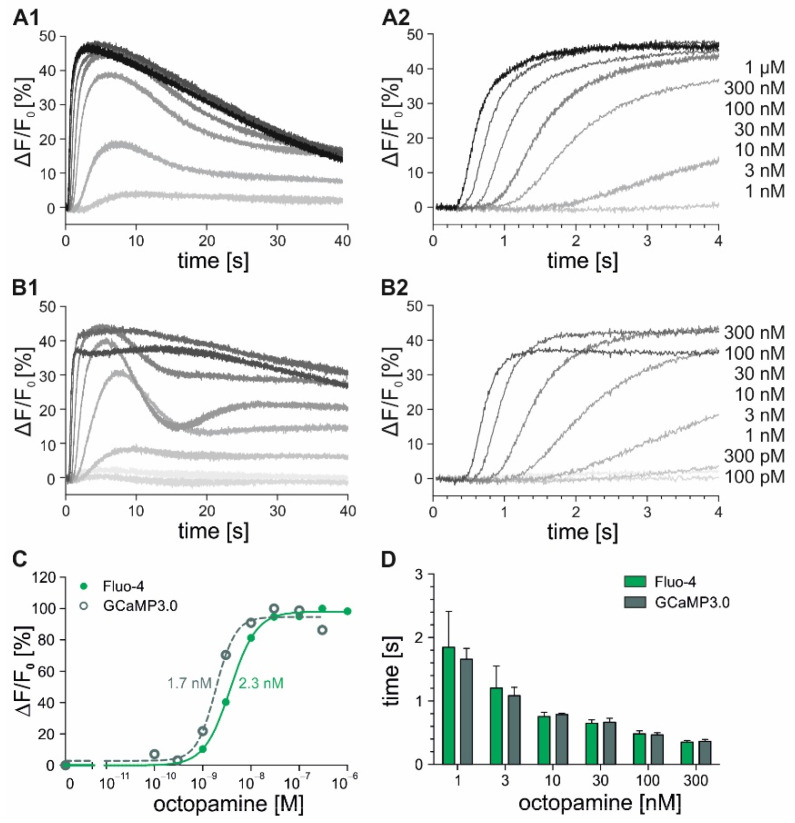
Stopped-flow experiments with DmOctα1B- and DmOctα1B-GCaMP3.0-expressing cell lines. Fluo-4-loaded DmOctα1B (**A**) and DmOctα1B-GCaMP3.0 (**B**) cells were stimulated with increasing octopamine concentrations ranging from 10^−10^–10^−6^ M in stopped-flow measurements. Fluorescence was monitored over 40 s and fluorescence changes (ΔF/F_0_) for each octopamine concentration were calculated and plotted against the time (**A1**,**B1**). To resolve the initial signal response times, the first 4 s of each measurement were analyzed and are shown (**A2**,**B2**). Representative measurements from three independent datasets are shown. Each data point was obtained from triplicate measurements; (**C**) Concentration–response curves were established by plotting maximal changes in fluorescence against octopamine concentrations. Maximal changes in fluorescence ((F_max_ − F_0_)/F_0_ = ΔF/F_0_) at the highest octopamine concentration were normalized to 100% and EC_50_ values were obtained from nonlinear fitting of the data using GraphPad Prism v5.04. A representative concentration–response curve is shown. Mean EC_50_ values are indicated from three independent datasets; (**D**) Bar graph indicating the time (s) passed until fluorescence signals were detected with Fluo-4-loaded (green) and GCaMP3.0-expressing (grey) cells (y-axis) and plotted against octopamine concentrations. Mean values ± SEM from three independent datasets are shown.

As described for the fluorimetric measurements performed in 96 well plates, the stimulation of the DmOctα1B receptors with octopamine results in an efflux of Ca^2+^ from the intracellular stores, which was now analyzed by stopped-flow experiments with high temporal resolution. The cells loaded with Fluo-4 or constitutively expressing GCaMP3.0 were diluted in extracellular solution (ES) to a density of 1 × 10^6^ cells/mL prior to application to the stopped-flow system. The cells were mixed with increasing ligand concentrations and the fluorescence signals were monitored for 40 s (Figure 3). The increasing ligand concentrations lead to a rise in the fluorescence intensities. The initial Ca^2+^ responses were detected at 1 nM octopamine and saturated at ligand concentrations ≥ 30 nM. After reaching a maximum, the fluorescence intensities decreased (Figure 3A1). This effect was more pronounced for concentrations ≤ 30 nM. In the cells expressing GCaMP3.0 (DmOctα1B-GCaMP3.0), additional increases in Ca^2+^ were resolved (Figure 3B1), probably monitoring the Ca^2+^ oscillations that are known to be induced upon DmOctα1B activation [21,33]. To determine the ligand affinities for the receptor, the maximal fluorescence intensities were plotted against the octopamine concentrations. This resulted in the concentration–response curves depicted in Figure 3C. The EC_50_ values were obtained from nonlinear fitting of the data, using GraphPad Prism v5.04. With 2.3 nM for the Fluo-4-loaded and 1.7 nM for the GCaMP3.0-expressing cells, the EC_50_ values were very similar. Notably, these values were about one order of magnitude lower than those determined with fluorimetric measurements in 96 MWPs (see Table 1). Besides the determination of the EC_50_ values, information about reaction velocities could be gained when analyzing the first 4 s of the measurements. The increasing octopamine concentrations correlated with faster signal detection (Figure 3A2,B2). The absolute response times (Figure 3D) showed that low ligand concentrations ≤ 3 nM (~EC_50_ value) lead to rather slow responses ~2 s. In contrast, the saturating octopamine concentrations (≥30 nM) resulted in fast response times of ~300 ms. The reaction velocities in a concentration range from 3 nM to 300 nM octopamine were almost identical for the Fluo-4-loaded and the GCaMP3.0-expressing cells.

### 2.3. Time-Resolved Measurements with DmOctβ1-Expressing Cells

**Figure 4:**
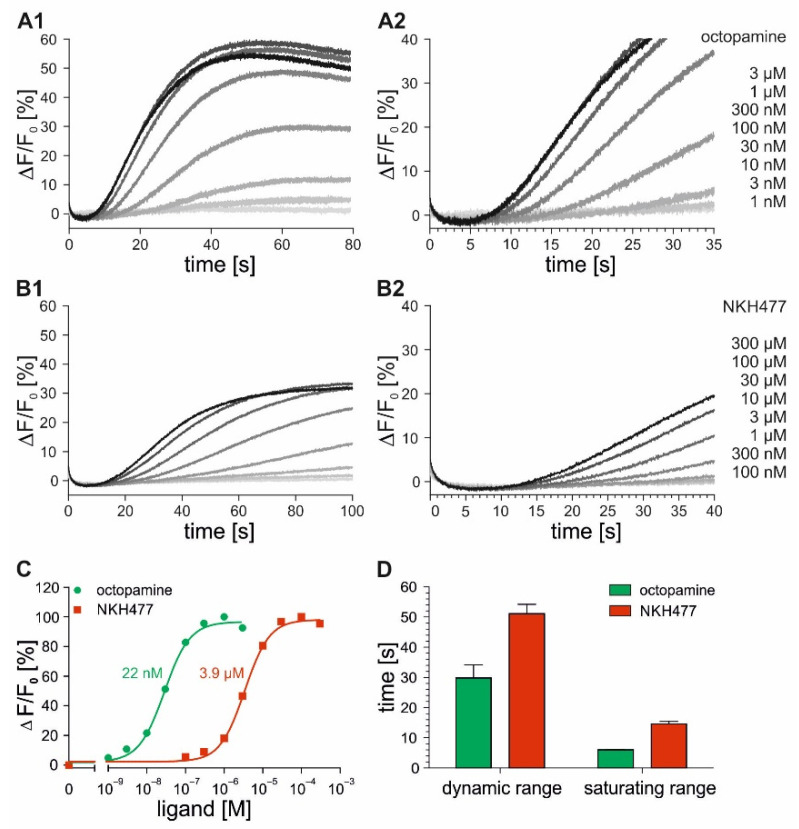
Stopped-flow experiments with flpTM-DmOctβ1 cells loaded with Fluo-4. Fluo-4-loaded flpTM-DmOctβ1 cells were stimulated in stopped-flow experiments with increasing octopamine (**A**) and NKH477 (**B**) concentrations. Fluorescence intensities were monitored over 80 s and 100 s, respectively. Fluorescent changes (ΔF/F_0_) for each octopamine and NKH477 concentration were calculated and plotted over time (**A1**,**B1**). To resolve signal response times, the initial 35 s and 40 s of each measurement are displayed (**A2**,**B2**). Shown are representative measurements from three independent datasets. Each data point was obtained from triplicate measurements; (**C**) Concentration–response curves were generated by plotting maximal changes in fluorescence against octopamine (green) and NKH477 (red) concentrations. Maximal changes in fluorescence at the highest ligand concentrations were normalized to 100% and EC_50_ values were obtained from nonlinear fitting of the data using GraphPad Prism v5.04. Shown is a representative concentration–response curve from three independent datasets. Mean EC_50_ values are indicated; (**D**) Bar graph showing the time until signal was detected at concentrations in the range of EC_50_ values (dynamic range) and at saturating concentrations (saturating range) for octopamine (green) and for NKH477 (red). Each data point was obtained from triplicate measurements. Shown are mean values ± SEM from three independent datasets.

In successive stopped-flow measurements, the flpTM-DmOctβ1 cells loaded with Fluo-4 were stimulated with increasing octopamine (1 nM–3 µM) and NKH477 (100 nM–300 µM) concentrations. The fluorescence intensities were monitored over 80 s and 100 s, respectively (Figure 4A1,B1). The fluorescence intensities increased and finally reached a plateau with increasing concentrations of both of the ligands. However, the saturating ligand concentrations differed strongly between octopamine (≥300 nM) and NKH477 (≥100 µM). By plotting the maximal changes in fluorescence (ΔF/F_0_) against ligand concentrations, concentration–response curves were generated and EC_50_ values were calculated (Figure 4C). The EC_50_ values obtained for NKH477 (3.9 µM) were about two orders of magnitude higher than those for octopamine (22 nM). As observed for the DmOctα1B cell lines (see Figure 3D), the signal response times of flpTM-DmOctβ1 cells were faster at higher ligand concentrations (Figure 4A2,B2). In Figure 4D, the time until the signals were detected (for details, see Materials and Methods, Section 4.4) are plotted for the ligand concentrations covering the calculated EC_50_ value (dynamic range) and for saturating concentrations (saturating range). The signal response times of the flpTM-DmOctβ1 cells for octopamine were in the range of 30 s (dynamic range) and 6 s (saturating range), whereas the signal detection upon NKH477 application was observed after 50 s (dynamic range) and 16 s (saturating range). The signal response times were about three- to five-fold faster at the saturating concentrations in comparison to the octopamine and NKH477 concentrations in the dynamic range.

**Figure 5:**
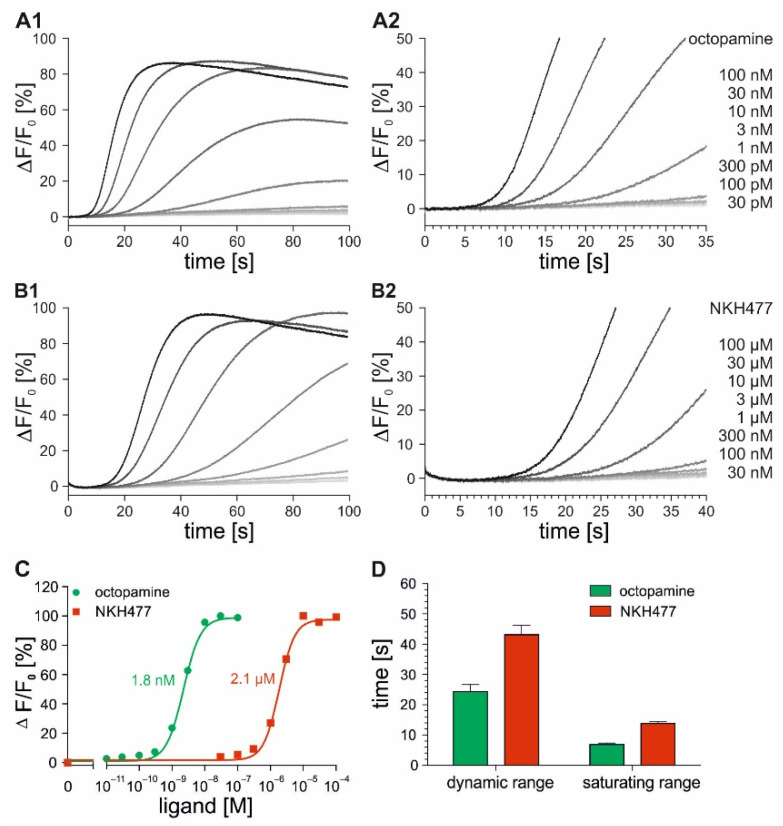
Stopped-flow experiments with FlpTM-DmOctβ1-GCaMP3.0 cell lines. FlpTM-DmOctβ1-GCaMP3.0 cells were stimulated with increasing octopamine (**A**) and NKH477 (**B**) concentrations. Fluorescence intensities were monitored over 100 s. Fluorescence changes (ΔF/F_0_) for each octopamine and NKH477 concentration were calculated and plotted over time (**A1**,**B2**). To resolve signal response times, the initial 35 s and 40 s of each measurement are shown (**A2**,**B2**). Shown are representative measurements from three independent datasets. Each data point was obtained from triplicate measurements; (**C**) Concentration–response curves were generated by plotting maximal changes in fluorescence against octopamine (green) and NKH477 (red) concentrations. Maximal changes in fluorescence at the highest ligand concentrations were normalized to 100% and EC_50_ values were obtained from nonlinear fitting of the data using GraphPad Prism v5.04. A representative concentration–response curve from three independent datasets is shown. Mean EC_50_ values are indicated; (**D**) Bar graph showing the time until signal was detected at concentrations in the range of EC_50_ values (dynamic range) and at saturating concentrations (saturating range) for octopamine (green) and for NKH477 (red). Each data point was obtained from triplicate measurements. Shown are mean values ± SEM from three independent datasets.

As described for the Fluo-4-loaded flpTM-DmOctβ1 cells, similar experiments were performed with the flpTM-DmOctβ1-GCaMP3.0 cells (Figure 5). The cells were successively mixed with increasing concentrations of octopamine (30 pM–100 nM) or NKH477 (30 nM–100 µM) and the fluorescence intensities were recorded over 100 s. For both of the ligands, increasing ligand concentrations led to an increase in the fluorescence emission finally reaching a plateau (Figure 5A1,B1).

Concentration–response curves were generated and the EC_50_ values were determined. The EC_50_ values were 1.8 nM for octopamine and 2.1 µM for NKH477. Increasing the octopamine or NKH477 concentrations both resulted in faster signal detection (Figure 5A2,B2). In Figure 5D, the time until the signal onset is plotted for the ligand concentrations covering the calculated EC_50_ value (dynamic range) and for the saturating concentrations (saturating range). The signal response times of the flpTM-DmOctβ1-GCaMP3.0 cells for octopamine were approximately 24 s (dynamic range) and 6 s (saturating range), whereas the signal detection upon NKH477 application was observed after 40 s (dynamic range) and 13 s (saturating range). Table 2 summarizes the EC_50_ values and response times for DmOctα1B- and DmOctβ1-receptor-expressing cell lines obtained in stopped-flow measurements.

The EC_50_ values determined for the DmOctα1B receptor with octopamine were in the same, low nanomolar range in both of the Fluo-4- and GCaMP3.0-based measurements. In the DmOctβ1 receptor-expressing cells, the EC_50_ values differed depending on whether Fluo-4 (22 nM) or GCaMP3.0 (≈2 nM) was used for the detection. These results were reminiscent of those performed in the plate reader (see Table 1) where the EC_50_ values determined with Fluo-4 were higher than those determined with GCaMP3.0. In contrast, the activation of adenylyl cyclases with NKH477 in the DmOctβ1 receptor-expressing cells loaded with Fluo-4 or co-expressing GCaMP3.0 resulted in similar EC_50_ values in the low micromolar range. Notably, the signal response times at saturating ligand concentrations were almost identical for the experiments performed with Fluo-4 and GCaMP3.0 in DmOctα1B (0.35 s) or flpTM-DmOctβ1 (octopamine, 6 s; NKH477, 13 s) cell lines. However, the different signal response times in the flpTM-DmOctβ1 cell lines that were uncovered at ligand concentrations in the dynamic range with signal detection using GCaMP3.0 were faster (see Figure 4D and Figure 5D).

### 2.4. Time-Resolved Measurements with Caged Compounds

The caged compounds are photolabile, biologically inert variants of bioactive molecules. Once irradiated with light, the “cage” group is photolytically cleaved and the bioactive molecule is released. Fast kinetic measurements can be performed as the release of the active molecule occurs within milliseconds, leading to instant activation at sites which are otherwise difficult to access.

Here, the caged compounds were employed to further investigate the kinetics of the DmOctβ1 receptor-signaling in the flpTM-DmOctβ1 cells. To achieve the photolytic cleavage of the cage group, a UV-flash was triggered at a defined time point, upon which the ligand was released and could interact with its target. The DmOctβ1 receptor and the CNGA2 channel were addressed using coumarin-based caged compounds of octopamine [34] and cAMP [35], respectively.

The flash photolysis of the caged compounds was achieved with UV-light (wavelength 405 nm). Neutral density filters that regulate the transmission of light (5%, 25%, 50%, 80%, 100%) were installed, allowing application of different light intensities.

As high intensities of UV-light are typically required for the efficient release of the caged group, the potential secondary effects due to UV-light flashes on flpTM-DmOctβ1 and flpTM-DmOctβ1-GCaMP3.0 cells were assessed.

**Figure 6:**
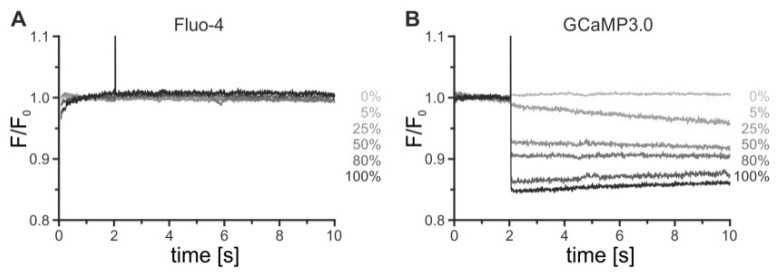
Effects of UV-light application on Fluo-4-loaded or GCaMP3.0-expressing cells. Fluo-4-loaded flpTM-DmOctβ1 (**A**) and flpTM-DmOctβ1-GCaMP3.0 cells (**B**) in extracellular solution were applied to the stopped-flow system and illuminated with a 50 ms UV-flash 2 s after the cuvette was completely filled. Fluorescence was monitored for 10 s. Successive measurements with increasing light intensities (0–100%) were performed. To visualize changes in fluorescence, the fluorescence intensity (F) was normalized to fluorescence intensity before UV-light application (F_0_) and F/F_0_ was plotted over time.

The FlpTM-DmOctβ1 cells, either loaded with Fluo-4 or constitutively expressing GCaMP3.0, were diluted in extracellular solution (ES) to a density of 1 x 10^6^ cells/mL. Stopped-flow experiments were performed, as described before. The cells were mixed in the cuvette with ES and 2 s after it was completely filled, a UV-flash was triggered for 50 ms and the fluorescence intensity was recorded. This was repeated in successive measurements using increasing light intensities (0–100%). To visualize the changes in fluorescence, the fluorescence intensity (F) was normalized to the fluorescence intensities recorded before the UV-flash (F_0_). The data (F/F_0_) were plotted over time for both the flpTM-DmOctβ1 and the flpTM-DmOctβ1-GCaMP3.0 cells (Figure 6).

In the flpTM-DmOctβ1 cells loaded with Fluo-4, the illumination did not cause any cellular response resulting in a Ca^2+^-dependent change of the dye’s photophysical properties. The fluorescence intensities before and after a UV-flash were identical for all of the light intensities. In contrast, the UV-light application to flpTM-DmOctβ1-GCaMP3.0 cells resulted in a strong decrease in fluorescence, which most likely was caused by bleaching of GCaMP3.0. Notably, the bleaching effect rose with the increasing light intensity. At 100% light intensity, the fluorescence intensity was reduced by about 15%. These results indicated that Fluo-4 but not GCaMP3.0 could be used in experiments to photolytically activate the caged compounds. Hence, all of the successive experiments with the caged compounds were performed in flpTM-DmOctβ1 cells, loaded with Fluo-4.

**Figure 7:**
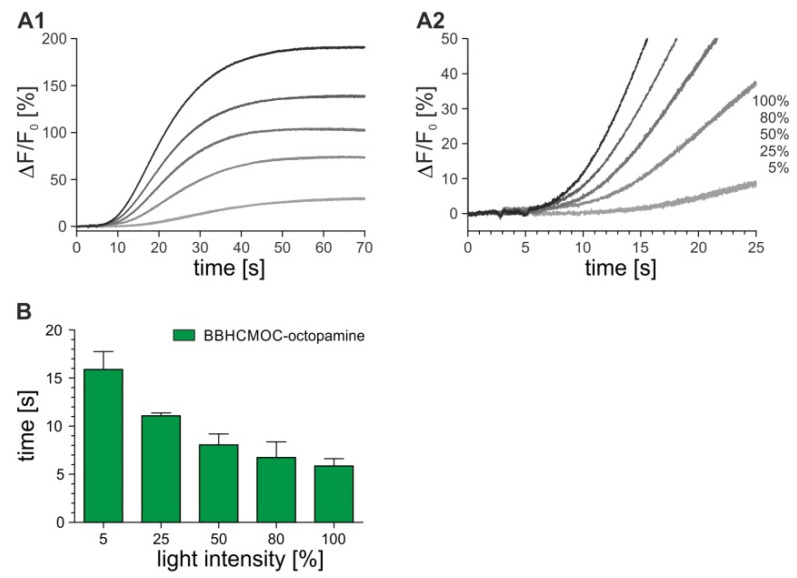
BBHCMOC-octopamine photolysis for activation of the DmOctβ1 receptor in flpTM-DmOctβ1 cells. (**A1**) flpTM-DmOctβ1 cells loaded with Fluo-4 were mixed in the cuvette of a stopped-flow system with a final concentration of 10 µM BBHCMOC-octopamine. After 2 s incubation a 50 ms UV-flash (405 nm) was triggered and fluorescence intensity was recorded for 70 s; (**A2**) Measurements were repeated applying different light intensities (5–100%). The time point of triggering the light flash was defined as t = 0. The first 25 s of the reaction were plotted. A representative measurement from two independent datasets is shown; (**B**) Signal response times ± SD obtained from two independent measurements were plotted against light intensities (%).

In the previous stopped-flow experiments, we initiated the DmOctβ1 receptor-activated reaction cascade by mixing cells with increasing concentrations of octopamine. Now, we used a similar strategy but substituted the biogenic amine for a constant concentration of octopamine fused to a coumarin-based cage group ({8-[bis(carboxymethyl)aminomethyl]-6-bromo-7-hydroxycoumarin-4-yl}methoxycarbon-yl; BBHCMOC-octopamine) [34] from which the biologically active octopamine was released by light flashes. The Fluo-4-loaded flpTM-DmOctβ1 cells were mixed with a final concentration of 10 µM BBHCMOC-octopamine in the cuvette of the stopped-flow apparatus. After 2 s, a UV-flash was triggered for 50 ms and the fluorescence intensities were recorded. The time when triggering the light flash was defined as time point t = 0. Different light intensities were successively applied (5%, 25%, 50%, 80%, 100% intensity). To avoid fluctuations in the [cAMP]_i_ due to cell-endogenous phosphodiesterase activity, the experiments were performed in the presence of 100 µM IBMX. Figure 7 summarizes the results that were obtained with BBHCMOC-octopamine.

The photolysis of BBHCMOC-octopamine with increasing light intensities resulted in increasing changes in fluorescence (ΔF/F0). The photochemical quantum yield for BBHCMOC-octopamine was determined as 0.11 in a previous study [34]. Thus, approximately 1 µM octopamine could be released from 10 µM BBHCMOC-octopamine. In the stopped-flow experiments described above, 1 µM octopamine was already found to induce saturating Ca^2+^ responses in flpTM-DmOctβ1 cells (see Figure 5A,B). This finding was also corroborated by the experiments with BBHCMOC-octopamine (Figure 7A). The successive UV-flashes of the same intensity in one stopped-flow measurement did not further increase the Ca^2+^ responses. The response times, however, correlated nicely with the increasing light intensities and thus the increasing octopamine concentrations (Figure 7B). The response times ranged from about 16 s at low octopamine concentrations (5% light intensity) to 6 s at high concentrations (100% light intensity). In summary, the signal response time of 6 s using caged octopamine nicely matched the results that were obtained with non-caged octopamine (see Table 2) on the Fluo-4-loaded flpTM-DmOctβ1 cells.

As an integral component of the flpTM-DmOctβ1 cell line, the cAMP-sensitive CNG channel (flpTM; see [32]) enables the detection of cellular cAMP dynamics via mediating cAMP-dependent opening of the channel pore and resulting in Ca^2+^ influx. Hence, we applied caged cAMP to determine the time elapsing from photolytic cAMP release to Ca^2+^ detection. A (7-diethylaminocoumarin-4-yl)methyl (DEACM)-modified [35] cAMP was used. The FlpTM-DmOctβ1 cells were loaded with Fluo-4 and DEACM-cAMP. For the stopped-flow measurements, the cells were mixed with ES in the cuvette and after 2 s, a UV-flash was triggered for 10 ms to release cAMP. The fluorescence signals were monitored for 12 s. The time point when triggering the light flash was defined as t = 0. Different concentrations of DEACM-cAMP were used (10 µM, 30 µM, 50 µM, and 100 µM) for loading cells. Concentrations of 10 µM and 30 µM DEACM-cAMP resulted in the stable fluorescence signals and provided information about the time elapsed from cAMP release to Ca^2+^ detection. In successive experiments, different light intensities were applied (5%, 25%, 50%, 80%, 100%). The experiments were performed in the presence of 100 µM IBMX to avoid fluctuations in cAMP due to cell-endogenous phosphodiesterases.

**Figure 8:**
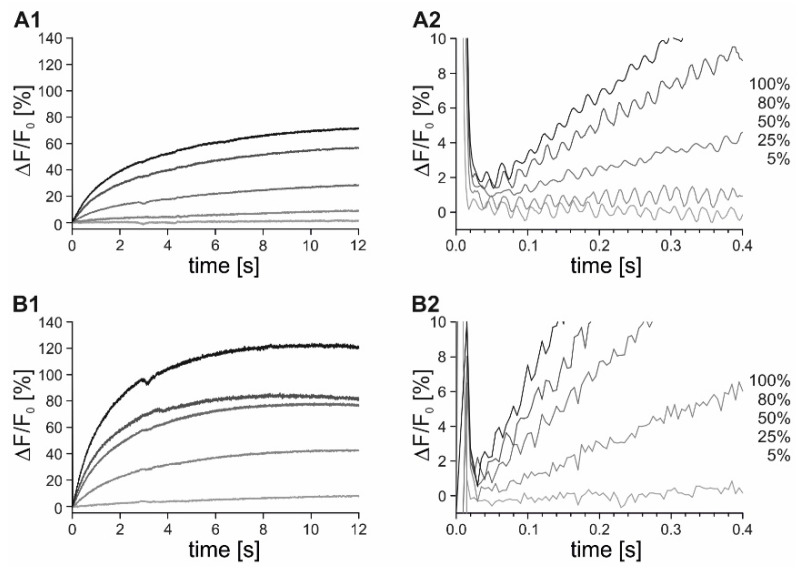
Photolysis of DEACM-cAMP for activating the CNG channel in flpTM-DmOctβ1 cells. FlpTM-DmOctβ1 cells were loaded with Fluo-4 and 10 µM (**A**) or 30 µM (**B**) DEACM-cAMP, respectively. Cells were mixed in a cuvette of a stopped-flow system with ES. After 2 s incubation a 10 ms UV-flash was triggered and the fluorescence intensity was recorded for 12 s (**A1**,**B1**). Measurements were repeated applying different light intensities (5–100%). The time point of triggering the light flash was defined as t = 0. To gain detailed temporal information on signal onset, the initial 0.4 s of the reactions was plotted (**A2**,**B2**).

In cells either loaded with 10 µM or 30 µM DEACM-cAMP, the photolysis of the caged compound with increasing light intensities correlated with increasing changes in fluorescence (ΔF/F_0_; Figure 8A1,B1). The photochemical quantum yield for the DEACM-cAMP was determined as 0.21 in a previous study [35]. Based on this data, approximately 2 µM or 6 µM cAMP was released from 10 µM and 30 µM DEACM-cAMP, respectively. From electrophysiological experiments, it was known that the EC_50_ for opening the CNG channel (A2 homomer; Ludwig et al., 1990) was ≈2 µM cAMP.

The calcium signals occurred with a slight delay after photolysis in the cells loaded with 10 µM DEACM-cAMP. At 25% light intensity, only a slight and slow increase in fluorescence was detected. In contrast, a strong rise in the fluorescence was observed approximately 60 ms after the light flash for light intensities of 80% and 100% (Figure 8A2), indicating that binding of cAMP to the CNG channel, influx of Ca^2+^, and detection of Ca^2+^ by Fluo-4 happens within this time frame. Notably, in the cells loaded with 30 µM the DEACM-cAMP, Ca^2+^ signals occurred immediately after the light flash and, therefore, the signal response times could hardly be resolved (Figure 8B).

In summary, the stopped-flow experiments revealed that the cellular signaling processes via DmOctβ1 receptors might take up to 6 s, independently of whether the activator is released from a caged compound or applied as a ligand solution. The time course was not dominated by the read-out component, i.e., the cAMP-gated CNG channel. Activating the last step in the entire signaling cascade took ≈60 ms. Thus, the remaining steps from receptor activation, interaction with stimulatory G-proteins, stimulation and production of cAMP by endogenous adenylyl cyclases add up to a total of ≈5.9 s. In contrast, with ≈360 ms, signals induced by the DmOctα1B receptor, coupling via phospholipase C to IP_3_-mediated Ca^2+^-release from intracellular stores was much faster, although a similar sequence of participating cellular components were engaged.

## 3. Discussion

In recent years, several techniques were developed to investigate the kinetics of GPCR-mediated signaling. Numerous studies examined ligand binding, receptor activation, G-protein coupling, and G-protein activation. Frequently inter- and intramolecular events were monitored with Förster resonance energy transfer (FRET), employing the labelling of individual interaction partners with suitable FRET donors and acceptors [36,37,38]. Due to the fact that only two interaction partners can be monitored at once, the investigation of entire signaling cascades is not possible. Furthermore, experiments are restricted to single cell applications and typically require high resolution microscopy analyses.

### 3.1. Functional and Pharmacological Characterization of Transgenic Cell Lines

Here, we applied multicellular approaches at different levels of temporal resolution to gain insight into the cellular Ca^2+^ and cAMP signaling pathways activated by two homologous biogenic amine receptors. The cell lines that constitutively express the octopamine receptors that transduce extracellular signals via the second messengers Ca^2+^ (DmOctα1B; [20,21]) or cAMP (DmOctβ1; [21,22]) were employed. The changes in [Ca^2+^]_i_ were monitored with the Ca^2+^-sensitive dye Fluo-4 [29] or with the genetically encoded Ca^2+^ indicator GCaMP3.0 stably transfected into the cell lines [30]. The cells equipped with DmOctβ1 receptors were additionally transfected with a cAMP-sensitive variant of the bovine olfactory CNG channel [31,32]. The binding of cAMP to, and opening of, the CNG channels results in an influx of Ca^2+^ into the cells. These Ca^2+^ signals were monitored either via Fluo-4 or in cells that were also stably transfected with the gene encoding GCaMP3.0.

In order to set the framework, all of the cell lines were initially characterized in multicellular fluorimetric measurements in a plate reader. As frequently applied in medium to high throughput assays, the experiments were performed in 96 multi well plates in a fluorescence reader and the EC_50_ values for octopamine and NKH477 were determined. As summarized in Table 1, the EC_50_ values for octopamine on DmOctα1B receptors were very similar when the Ca^2+^ signals were registered with Fluo-4 or GCaMP3.0 (12 nM and 14 nM). The EC_50_ values obtained for DmOctβ1R were even lower for octopamine (1.2 nM, Fluo-4; 0.4 nM, GCaMP3.0). Since DmOctβ1R stimulates the adenylyl cyclases via G_s_ G-proteins, the direct activation of these enzymes was examined with NKH477, a water-soluble forskolin derivative. Similar to the results in a previous study [32], the EC_50_ values were in the micromolar range (4.5 µM, Fluo-4; 0.1 µM GCaMP3.0).

A central aim of our investigation was to gain a temporal insight into the individual steps of GPCR-induced signaling cascades. For this purpose, stopped-flow measurements were applied. Stopped-flow experiments allow the investigation of biochemical processes with millisecond time resolution [39,40]. In recent years, stopped-flow measurements have been successfully applied to analyze, e.g., the kinetics of signaling processes in spermatozoa [41]. Here, we adapted the stopped-flow measurements to stably transfected HEK293 cell lines. An important step was to define a decent cell density and flow rate supporting both the short dead times to signal onset (here 36 ms) in combination with good cell-survival rates (≈95%). This goal was achieved with cell suspensions containing 1 × 10^6^ cells/mL and flow rates of 1 mL. Almost identical EC_50_ values for octopamine on DmOctα1B-expressing cells were obtained in stopped-flow measurements when the Ca^2+^ signals were measured with Fluo-4 (2.3 nM) or GCaMP3.0 (1.7 nM). In addition, the EC_50_ values for NKH477 on flpTM-DmOctβ1 cells with 3.9 µM and 2.1 µM for Fluo-4 and GCaMP3.0, respectively, were rather similar. However, the EC_50_ values for octopamine on flpTM-DmOctβ1 cells differed by about one order of magnitude (Fluo-4: ≈22 nM, GCaMP3.0: ≈1.8 nM). All of the EC_50_ values obtained in fluorimetric measurements in 96 multi well plates and in stopped-flow experiments are summarized in Table 3.

Although there is no final answer, yet, as to why there were some differences comparing both measuring regimes, reasonable explanations might be: (a) in Ca^2+^ fluorimetric measurements in a plate reader, the cells are attached to the bottom of the well. The diffusion of the ligand to the adherent cells might be rate-limiting. Typically, such measurements are examined once a steady state has been reached. In a stopped-flow system, the cell suspension is rapidly mixed with the ligand-containing solution, leading to faster dwell times between the receptor and ligand and, thus, resulting in faster signal onset; (b) when using Fluo-4 as a Ca^2+^ indicator, the cells have to be incubated in dye-containing solution to achieve uptake of the dye. However, the intracellular Fluo-4 concentration cannot be reliably adjusted. In contrast, the cell lines constitutively expressing GCaMP3.0 typically display a homogeneous expression level and cellular distribution of the sensor protein that eventually leads to more consistent results.

### 3.2. Time-Resolved Measurements of Signaling Cascades

Due to the short dead time (36 ms) of the stopped-flow system upon mixing of the reactants, it was possible to gain information about the time dependence of the signal output originating from the DmOctα1B- or DmOctβ1 receptor activation. For the DmOctα1B-expressing cells, the speed of the reaction correlated with the octopamine concentrations. A rather slow signal detection (2 s) was observed with 1 nM octopamine, whereas fast responses of ≈350 ms were measured with 300 nM octopamine. No difference was observed when Fluo-4 or GCaMP3.0 was used for the Ca^2+^ detection. A concentration dependence of response times was also observed for the flpTM-DmOctβ1-expressing cell lines for octopamine, as well as for NKH477. The response times at the saturating concentrations were similar for both Fluo-4 and GCaMP3.0 (6 s, octopamine; 13 s, NKH477) but slightly differed at the concentrations in the dynamic range (24 s, octopamine; 40 s, NKH477) with faster signal detection occurring with GCaMP3.0. The dissociation constants (K_d_) for Ca^2+^ were determined in vitro and are in a similar range for Fluo-4 (345 nM; [29]) and GCaMP3.0 (840 nM and 405 nM; [30,42]). Nevertheless, some technical drawbacks of Fluo-4, such as the necessity to incubate live cells in dye-containing solution in advance of the experiment for 90 min, the variability of its intracellular concentration, and a slightly lower Ca^2+^ affinity of GCaMP3.0, might culminate in more dynamic signal detection with the GECI. Although addressing the same signaling pathway in the flpTM-DmOctβ1 cells, there was a clear difference in the response times at saturating ligand concentrations for octopamine with 6 s and for NKH477 with 13 s. This result is most likely due to the fact that for the activation of adenylyl cyclases, NKH477 must bind intracellularly to the catalytic domains [43]. Thus, after mixing the cell suspension and ligand solution, the compound first must cross the plasma membrane to reach its target, resulting in a prolonged reaction time. In order to overcome these limitations, a caged derivative of NKH477 would be an alternative but, unfortunately, this is not commercially available at present.

However, we could make use of two caged compounds, i.e., BBHCMOC-octopamine [34] and DEACM-cAMP [35], to access the starting as well as the exit point in the signaling cascades. Both of the compounds are rendered biologically inactive by chemical modification with a light-sensitive protection group. After photolytic cleavage of the cage group, the bioactive molecule is released within milliseconds, allowing the instant activation of target structures [44]. Prior to the application of the caged compounds it was tested whether UV-light (405 nm) had any effect on Fluo-4-loaded or GCaMP3.0-expressing cells. A significant bleaching of GCaMP3.0, probably due to a broader excitation spectrum of the GECI compared to Fluo-4 [30], was detected. Therefore, all of the measurements with caged compounds were performed in the Fluo-4-loaded cells.

In the flpTM-DmOctβ1 cells, the signal response times correlated with applied light intensities, i.e., active ligand concentrations. The maximal response times obtained upon photolysis of BBHCMOC-octopamine were identical with those obtained with non-modified octopamine (≈6 s). This confirmed the experimental rationale, as octopamine binds to the receptor extracellularly and, therefore, activation occurs instantaneously upon mixing the cells with non-modified octopamine or after photolytic cleavage of BBHCMOC-octopamine. Furthermore, the experiments with caged cAMP were performed to obtain the data for the time interval from cAMP release to Ca^2+^ influx through CNG channels in the plasma membrane. At high concentrations (30 µM DEACM-cAMP), the Ca^2+^ signals were detected immediately after photolysis. At a lower concentration (10 µM DEACM-cAMP), the first Ca^2+^ signals occurred approximately 60 ms after photolysis. Thus, CNG channel activation, influx of Ca^2+^, and detection of Ca^2+^ occurs within ≤60 ms, implying that the DmOctβ1 receptor-, G_αs_-, and adenylyl cyclase-activation, as well as the cAMP synthesis, takes a total of ≈5.9 s. In comparison, the reaction cascade induced by the DmOctα1 receptor operated at a significantly faster time scale (≈360 ms).

In recent years, several groups have contributed to unraveling the kinetics of the GPCR activation, mainly addressing ligand binding-, receptor activation-, receptor to G-protein coupling-, and G-protein activation processes. Most of the studies used inter- and/or intramolecular FRET measurements. The receptor activation, monitored via conformational changes of the transmembrane domains, was reported to occur within 30–50 ms [45,46,47,48,49,50]. Recent investigations, however, employing molecular dynamics simulations imply that the ligand binding and initial conformational changes of a GPCR occur within microseconds [51,52,53,54]. The receptor to the G-protein interaction occurred within 30–50 ms [55], indicating rapid activation kinetics, although other studies indicated that G-protein activation is about ten times slower than receptor activation with time constants of 300–500 ms; this might be due to the rate-limiting step of the GDP/GTP exchange in the α subunits of these trimeric G-proteins [47,55,56,57]. The activated G-proteins then interacted with the downstream effectors, such as ion channels and/or enzymes. Whereas the G-protein activated the inwardly rectifying potassium (GIRK) channels open within 200–500 ms after receptor activation [58], the cAMP responses may show a lag time of several seconds [59,60,61].

These findings indicate that the kinetics of receptor activation might be well-conserved among the GPCRs. The entire signaling cascades, however, differ significantly, and largely depend on the properties of downstream effectors. This might also explain the strong difference in the signal response times between the cells expressing DmOctα1B or DmOctβ1 receptors. The DmOctα1B receptor activation initiates the PLC-mediated cleavage of phosphatidylinositol 4,5-bisphopsphate (PIP_2_) to IP_3_, which in turn causes the opening of the ligand-gated IP_3_-receptors in the endoplasmic reticulum and the Ca^2+^-influx into the cytoplasm. With ≥5000 molecules IP_3_ s^−1^ [62], the turnover number of phospholipase C is high. The affinity of the IP_3_ receptors was determined to be in the low nanomolar to picomolar range [63]. Considering the volume of a typical HEK293 cell with ≈1 pL (www.bioNumbers.org, accessed on 15 June 2022), the PLCs in the HEK293 cells generate sufficient IP_3_ to support activation of IP_3_ receptors in 100–200 ms. Considering 50 ms each, for the receptor activation and G-protein interaction, and ca. 200 ms for the IP_3_ synthesis, this calculated value nicely fits the experimental data obtained from the stopped-flow experiments on DmOctα1B-expressing cells. The situation in the DmOctβ1 receptor-expressing cells is different: (a) the affinity of the CNG channel (EC_50_ = 2µM) is lower than that of IP_3_-receptors and, consequently, requires a higher ligand concentration for activation; and (b) the turnover number of the adenylyl cyclases is much lower (maximal value 100 cAMP/s) [64,65,66] than that of the PLCs. Thus, both of the factors contribute to the longer response times that we have observed in the stopped-flow measurements.

In summary, we have shown that: (a) genetically modified cell lines can be successfully used for stopped-flow experiments to uncover the individual steps of signaling cascades at ms timescales; (b) Ca^2+^-imaging with either synthetic dyes or genetically encoded Ca^2+^ indicators is well suited to study even the different GPCR-mediated signaling pathways; and (c) caged compounds are a promising route to address and manipulate the signaling cascades at decisive points. Once the caged-NKH477 is available, this will allow us to finally examine the rate-limiting step in DmOctβ1 receptor signaling with high temporal accuracy.

## 4. Materials and Methods

### 4.1. Generation of Stably Transfected HEK293 Cell Lines

In this study, we made use of previously established stably transfected cell lines [21]. Briefly, human embryonic kidney cells (HEK293; #85120602, ECACC, Porton Down, Salisbury, UK) were transfected with 10 µg of DmOctα1B receptor-encoding construct by a modified calcium phosphate method [67]. The cell line constitutively expressing the DmOctα1B receptors was propagated in MEM+Glutamax medium containing 10% (v/v) fetal calf serum, 1% (v/v) non-essential amino acids, 1% (v/v) antibiotics/antimycotics, and 800 µg/µL Geneticin (all obtained from ThermoFisher Scientific/Gibco; Dreieich, Germany). A related human embryonic kidney 293-based cell line (HEK293; flpIn cells; Invitrogen/ThermoFisher Scientific; #750-07) that was transfected with a gene encoding a variant of the A2-subunit of the olfactory cyclic nucleotide-gated ion channel (CNG; [31]; flpTM cells, provided by Sibion Biosciences, Jülich, Germany) was transfected with 10 µg of DmOctβ1 receptor-encoding construct. The cell line constitutively expressing the CNG channels and DmOctβ1 receptors was named flpTM-DmOctβ1 and propagated in DMEM+Glutamax medium containing 10% (v/v) fetal calf serum, 1% (v/v) penicillin/streptomycin, and 100 µg/mL Hygromycin, as well as 800 µg/µL Geneticin (all obtained from ThermoFisher Scientific/Gibco). In order to overcome loading cells with the Ca^2+^-sensitive dye Fluo-4, both of the cell lines were additionally transfected with a plasmid encoding GCaMP3.0 in pcDNA6/myc-His A vector (Life Technologies/Thermo Fisher Scientific, Darmstadt, Germany). The stably transfected cell lines, DmOctα1B-GCaMP3.0 or flpTM-DmOctβ1-GCaMP3.0, were propagated in media as mentioned before but with Blasticidin (10 µg/mL) added to sustain the selection pressure on the GCaMP3.0-encoding cassette.

### 4.2. Ca^2+^ Fluorimetry in Stably Transfected Cell Lines

To monitor the intracellular changes of Ca^2+^, the cells stably transfected with the genetically encoded calcium indicator GCaMP3.0 (Tian et al., 2009) or loaded with the calcium-sensitive fluorescent dye Fluo-4-AM (Life Technologies/Thermo Fisher Scientific) were analyzed in the plate reader FLUOstar Omega (BMG Labtech, Ortenberg, Germany). The excitation was set to 485 nm and the emission was registered at 510 nm. The cells were seeded in 96 multi well plates with densities from 8000–15,000 cells/well 24 h before the measurements and incubated at 37 °C, 5% CO_2_, and 95% relative humidity. The cells that did not express GCaMP3.0, were incubated with 50 µL loading buffer for 90 min at room temperature in the dark. The loading buffer consisted of extracellular solution (ES = 120 mM NaCl, 5 mM KCl, 2 mM MgCl_2_, 2 mM CaCl_2_, 10 mM HEPES, 10 mM Glucose, pH 7.4 (NaOH)) supplemented with 1.7 µM Fluo-4-AM, 3 mM probenecid, and 0.02% (w/v) Pluronic F-127 (Sigma-Aldrich/Merck, Darmstadt, Germany). The loading buffer was removed and each well was filled with 90 µL ES. For the cell lines expressing DmOctβ1 receptor, ES was supplemented with 100 µM isobutyl-methylxanthine (IBMX; Sigma-Aldrich/Merck). The plates were transferred to the plate reader and the baseline fluorescence was recorded until the fluorescence had reached a stable value in each well (basal fluorescence, F_0_). Concentration series of ligands were applied (10 µL/well) and changes in fluorescence were recorded automatically. The concentration–response curves were established from at least three independent experiments with four-to-eight-fold determinations for each data point. The relative change in the fluorescence ((F_max_-F_0_)/F_0_) change was calculated and plotted against the ligand concentration. The data were analyzed and displayed (nonlinear regression plot, variable slope with four parameters) using Prism 5.04 software (GraphPad, San Diego, CA, USA).

### 4.3. Stopped-Flow Measurements with Live Cells

The stopped-flow measurements enable the analysis of biochemical processes with high temporal resolution, e.g., in a milliseconds to seconds time range. The method is based on the rapid mixing of two or more reactants simultaneously or with different time intervals. In stopped-flow measurements, the reactants are mixed in a mixing chamber and pushed into an observation chamber where the flow is stopped and the reaction is monitored. This can be completed optically, calorimetrically, or electrically. The time resolution is dependent on the flow rate (f) of the reactants as well as of the volume (v) that is needed to fill the mixing and reaction chambers. The time (t) that is needed until a reaction can be monitored or quenched, is calculated as follows: t = v/f. Variation of the flow rate and/or the volume allows for the analysis of the reactions at different time points.

For the stopped-flow measurements, the SFM-400 system (BioLogic, Münster, Germany) was used. The main component of the stopped-flow setup is the SFM-400 module with four 10 mL syringes, which can be filled by external valves. The syringes are driven by step motors. The reactants can be mixed in three individual mixing chambers (M1–M3). The volume between M1 and M2 is 21 µL, and 31 µL between M2 and M3. The observation chamber is a quartz cuvette (FC-15) with a reaction volume of 31 µL, installed behind M3. To avoid fluctuations of the reaction mixture in the cuvette, which might lead to noisy and inconsistent signals, a hard stop valve located behind the cuvette can be closed after injection of the reactants. The stopped-flow module is controlled via a MPS-60 control unit (BioLogic). Data acquisition and operation of the system were performed with the Bio-Kine 32 v4.74 software (BioLogic).

The reactions in the cuvette were analyzed via the changes in the fluorescence. A 150 W Xe/Hg-lamp (ALX-220; BioLogic) was used as a light source for the excitation. The excitation light was guided via a light guide towards the cuvette. To determine the wavelength of the excitation light, a 482 nm bandpass filter (482/35 nm BrightLine^®^ single-band bandpass filter; Semrock, Rochester, NY, USA) was used. A heat protection filter (3 mm; KG1, Schott; Mainz, Germany) was placed between the Xe/Hg-lamp and the excitation filter. The fluorescence was detected with a photomultiplier (PMS 200, BioLogic) installed rectangularly to the light path of the excitation light. Between the cuvette and the photomultiplier tube a 535 nm bandpass emission filter (XF3007 535AF35; Horiba, Oberursel, Germany) was placed to select for the emission light being detected by the photomultiplier. A voltage of 600 V was applied to the photomultiplier.

For the photolysis of the caged compounds with ultraviolet (UV)-light, a Xe-flash lamp (JML-C2; Rapp Opto Electronics, Wedel, Germany) was coupled to the cuvette via an UV-light permeable light guide (Schott). A UV-2 interference filter was placed between the Xe-flash lamp and light guide to select for the UV-light needed for the photolysis of the caged compounds. Additionally, it prevents the photomultiplier from being hit by light that is able to pass the emission filter. The maximal energy of one light flash at the surface of the cuvette was 107 mJ·cm^−2^ (set to C2 and 200 V). Neutral density (ND) filters (AHF Analysentechnik, Tübingen, Germany) could be installed between the interference filter and the Xe-flash lamp to regulate the intensity of the UV-light applied to the cuvette. To maximize the detection of the emission light, a test solution containing 50 nM of fluorescein in ES was added to the cuvette and the light guide was fixed to the Xe/Hg-lamp at a position where the photomultiplier detected maximal intensity.

The fluorescence measurements were performed either directly with the cells expressing GCaMP3.0 or after loading the cells with Fluo-4-AM. Changes in the intracellular Ca^2+^ were induced by stimulating the cells with a concentration series of different ligands. In the stopped-flow module, four different solutions can be mixed. Syringes S1–S4 inject the reactants into three different mixing chambers (M1–M3), with mixing chamber M3 leading to the cuvette. The time passing between the mixing of the reactants and their entry to the cuvette, where the reaction can be observed, is called dead time. For this time period, the reaction cannot be examined. The dead time is defined by two parameters: the velocity of the reaction mixture; and the volume that the reaction mixture needs to pass before entering the cuvette. To keep the dead time as small as possible, both of the reactants were mixed in M3. Syringes S3 and S4 were filled with the ligands diluted in ES and HEK293 cells diluted in ES, respectively. Syringes S1 and S2 were filled with ES. Whereas S2 was uncoupled from the system, S1 was used to wash the mixing chamber between measurements. The flow rate of the reaction mixture entering the cuvette was determined by the syringe program which was set with the Bio-Kine software. Each syringe program is divided into different phases, which defines the volume that is injected from a syringe into the corresponding mixing chamber.

In phase 1, the step motors of the syringes are synchronized and the signal detection is started. In phase 2, the system is washed with 400 µL ES to remove the remains of the previous measurement. ES stays in the system for 300 ms (phase 3). In phase 4, the actual measurement is started by the injection of the ligand and HEK293 cell suspension into the cuvette with a flow rate of 1.0 mL/s. This configuration, in combination with a flow rate of 1 mL, resulted in a dead time of 36 ms, i.e., the reactants have already reacted for 36 ms when entering the cuvette. The acquisition time was chosen depending on the velocity of the investigated reaction.

Prior to application to the system, the HEK293 cells grown on a 10 cm petri dish were washed with 5 mL PBS and scraped off in 2 mL PBS. The cells were pelleted (Sigma 3K12, 200 g, 22 °C, 5 min), re-suspended in ES and the cell number was determined. The cells constitutively expressing GCaMP3.0 were diluted to a density of 1 × 10^6^ cells/mL. The cells that did not express GCaMP3.0 were loaded with Fluo-4-AM in loading buffer (s.a.) for 45–90 min at 37 °C. To remove the loading buffer, the cells were centrifuged (Sigma 3K12, 200 g, 22 °C, 5 min) and re-suspended in ES to a density of 1 × 10^6^ cells/mL.

### 4.4. Data Analysis

**Figure 9:**
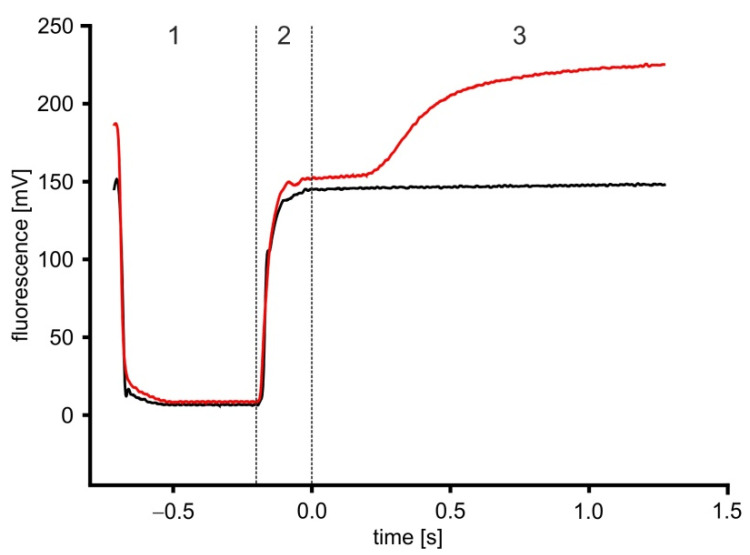
Diagram of typical stopped-flow fluorescence measurements. Cells were loaded with Fluo-4 and then either non-stimulated (black graph) or stimulated with ligand (red graph) submitted to the stopped-flow apparatus. Due to an initial washing step (1) with ES buffer, remaining fluorescence in the cuvette drops to almost 0 values. When Fluo-4 loaded cells are submitted during the stimulation phase (2) to the cuvette an increase in fluorescence reaching a plateau (=baseline) can be registered. Finally, during the measurement phase (3), typically stimulated cells showed a further increase in fluorescence. Due to the dead time of the system (36 ms), t = 0 was defined omitting the dead time.

Figure 9 shows the original data of stopped-flow measurements performed on unstimulated (black graph) and stimulated (red graph) HEK293 cells, loaded with Fluo-4. The fluorescence signals can be divided into three phases: washing phase (1); stimulation phase (2); and measurement phase (3). In the washing phase, the system is washed with ES buffer and the cells remaining in the cuvette from the previous measurement are removed. This leads to a sudden decrease in the fluorescence intensity when the cells are exchanged for ES. In the stimulation phase, the HEK293 cells and ligand are mixed in M3 and pushed into the cuvette, marked by an increase in fluorescence, which maximizes, once all of the ES from the washing phase is exchanged for cell suspension. Once the cuvette is completely filled with cell suspension and the flow is stopped by closure of the hard stop valve, the measurement phase starts.

Due to the dead time, the cells were already stimulated for 36 ms at the beginning of the measurement phase. Therefore, the dead time is subtracted to define the actual starting point (t = 0) of the reaction. At the beginning of the measurement phase, the signal generated by both the unstimulated and stimulated cells is rather similar. This is defined as baseline (F_0_). Then, the stimulated cells typically start showing an increase in fluorescence due to ligand activity. For analysis, the change in the fluorescence was calculated by subtracting the baseline value. The signals before t = 0 are not shown in graphs and the change in the fluorescence (ΔF = F_max_ − F_0_) is normalized and plotted as ΔF/F_0_ (%) against the ligand concentrations. Unless stated otherwise, the data were obtained from three–four successive measurements.

## Figures and Tables

**Table 1:** EC_50_ values of DmOctα1B and FlpTM-DmOctβ1 cell lines for octopamine and NKH477. EC_50_ values of DmOctα1B and FlpTM-DmOctβ1 cell lines for octopamine and NKH477 were determined with Fluo-4 and GCaMP3.0. Measurements with flpTM-DmOctβ1 cell lines were performed in the presence of 100 µM IBMX. EC_50_ values are from a typical experiment with eight-fold determinations for each data point.

	EC_50_/logEC_50_ ± SD	
Cell Line	Octopamine	NKH477
DmOctα1B	12.0 nM/−7.9 ± 0.1	n.d.
DmOctα1B-GCaMP3.0	14.0 nM/−7.8 ± 0.1	n.d.
flpTM-DmOctβ1	1.2 nM/−8.9 ± 0.04	4.5 µM/−5.34 ± 0.09
flpTM-DmOctβ1-GCaMP3.0	0.4 nM/−9.36 ± 0.04	0.1 µM/−6.91 ± 0.03

**Table 2:** EC_50_ values and signal response times at saturating octopamine and NKH477 concentrations are summarized for DmOctα1B- and flpTM-DmOctβ1-receptor-expressing cells loaded with Fluo-4 or constitutively expressing GCaMP3.0. Values represent mean ± SEM (triplicate determination for each data point) from three independent measurements.

	EC_50_		Signal Response Time	
Cell Line	Octopamine	NKH477	Octopamine	NKH477
DmOctα1B	2.25 ± 0.15 nM	-	0.35 ± 0.04 s	-
DmOctα1B-GCaMP3.0	1.70 ± 0.10 nM	-	0.36 ± 0.05 s	-
flpTM-DmOctβ1	22.00 ± 4.49 nM	3.92 ± 0.35 µM	6.00 ± 0.13 s	13.35 ± 0.82 s
flpTM-DmOctβ1- GCaMP3.0	1.78 ± 0.28 nM	2.12 ± 0.45 µM	6.00 ± 0.20 s	12.70 ± 0.52 s

**Table 3:** EC_50_ values obtained in fluorimetric measurements in 96 multi well plates and in stopped-flow experiments with DmOctα1B and flpTM-DmOctβ1 cell lines. EC_50_ values for octopamine and NKH477 were determined with Fluo-4-loaded or GCaMP3.0-expressing cell lines. Experiments with flpTM-DmOctβ1 and flpTM-DmOctβ1-GCaMP3.0 cells were performed in the presence of 100 µM IBMX.

	96 MWP		Stopped-Flow	
Cell Line	Octopamine	NKH477	Octopamine	NKH477
DmOctα1B	12.0 nM	-	2.3 nM	-
DmOctα1B-GCaMP3.0	14.0 nM	-	1.7 nM	-
flpTM-DmOctβ1	1.2 nM	4.5 µM	22.0 nM	4.0 µM
flpTM-DmOctβ1-GCaMP3.0	0.4 nM	0.1 µM	1.8 nM	2.0 µM

## Data Availability

Data and materials described in this study are available upon request from the corresponding author.

## Supplementary Materials

The following supporting information can be downloaded at: https://www.mdpi.com/article/10.3390/ijms23158516/s1.
