# Supplementary material for: Examination of Intracellular GPCR-Mediated Signaling with High Temporal Resolution
Source: Int J Mol Sci. 2022 Jul 31;23(15):8516. doi: 10.3390/ijms23158516 (PMC9369311; doi:10.3390/ijms23158516)
Supplement: Supplementary file 1 [file ijms-23-08516-s001.zip › ijms-1842772-supplementary.pdf]

Supplementary material

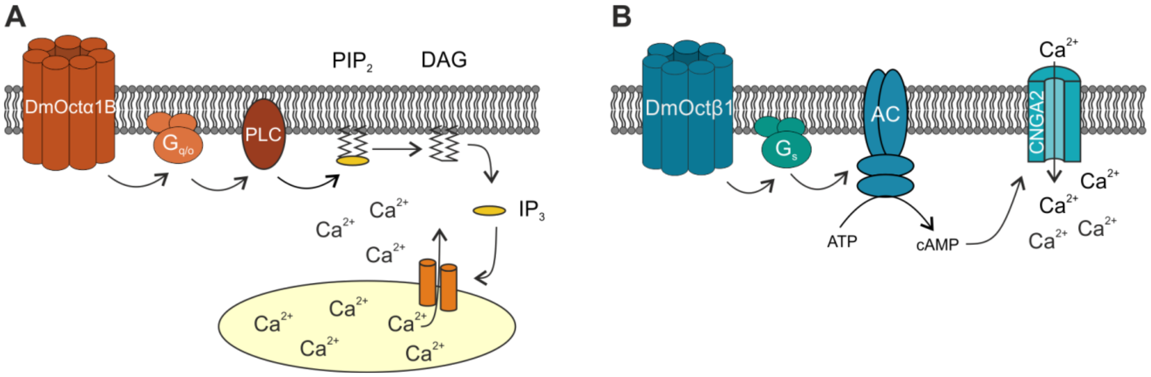

Figure S1: Protein composition of HEK293 cell lines used for kinetic measurement. (**A**) DmOctα1B cells: HEK293 cells constitutively expressing the octopamine receptor DmOctα1B. Stimulation of DmOctα1B leads to activation of a trimeric G-protein ($G_{q/o}$). Its α-subunit dissociates and stimulates phospholipase C which cleaves phosphatidylinositol 4,5-bisphosphate (PIP_2) into diacylglycerol (DAG) and inositol 1,4,5-trisphosphate (IP_3). IP_3 binds to ionotropic IP_3-receptors at the membrane of the endoplasmic reticulum and causes an efflux of Ca^{2+} into the cytoplasm. (**B**) FlpTM-DmOctβ1 cells: HEK293 cells constitutively expressing the octopamine receptor DmOctβ1 and a cAMP sensitive variant (T537M) of the CNGA2 ion channel. Stimulation of DmOctβ1 leads to activation of a trimeric G-protein (G_s). Its α-subunit stimulates endogenous adenylyl cyclase (AC) which catalyzes the synthesis of cAMP. Cyclic AMP binds to the CNGA2 channel leading to an influx of Ca^{2+} from the extracellular space. Main components of the signalling cascade can be stimulated individually with: octopamine/caged octopamine (->receptor), NKH477 (->AC), and caged cAMP (->CNGA2). Cell lines were loaded with Fluo-4 to monitor intracellular changes in Ca^{2+}. Additionally, both cell lines were stably transfected with the Ca^{2+}-sensitive fluorescence indicator GCaMP3.0.
